# Carbohydrate-Induced Insulin Signaling Activates Focal Adhesion Kinase: A Nutrient and Mechanotransduction Crossroads

**DOI:** 10.3390/nu12103145

**Published:** 2020-10-15

**Authors:** Dylan T. Wilburn, Steven B. Machek, Thomas D. Cardaci, Darryn S. Willoughby

**Affiliations:** 1 Exercise and Biochemical Nutrition Laboratory, Department of Health, Human Performance, and Recreation, Baylor University, Waco, TX 76706, USA; Dylan_Wilburn1@baylor.edu (D.T.W.); Steven_Machek2@baylor.edu (S.B.M.); tcardaci@email.sc.edu (T.D.C.); 2 Department of Exercise Science, Arnold School of Public Health, University of South Carolina, Columbia, SC 29208, USA; 3 School of Exercise and Sport Science, University of Mary Hardin-Baylor, Belton, TX 76513, USA

**Keywords:** focal adhesion kinase, insulin, crosstalk, mammalian target of rapamycin complex one (mTOR), insulin receptor substrate 1 (IRS-1), phosphatidylinositol 3-kinase (PI3K), mechanotransduction

## Abstract

Research has suggested that nutrient, exercise, and metabolism-related proteins interact to regulate mammalian target of rapamycin complex one (mTOR) post-exercise and their interactions needs clarification. In a double-blind, cross-over, repeated measures design, ten participants completed four sets to failure at 70% of 1-repitition maximum (1-RM) with 45 s rest on angled leg press with or without pre-exercise maltodextrin (2 g/kg) after a 3 h fast. Vastus lateralis biopsies were collected at baseline before supplementation and 1 h post-exercise to analyze Focal Adhesion Kinase (FAK), ribosomal protein S6 kinase beta-1 (p70S6K), insulin receptor substrate 1 (IRS-1), phosphatidylinositol 3-kinase (PI3K), and 5′ AMP-activated protein kinase (AMPK) activation. FAK and IRS-1 activity were only elevated 1 h post-exercise with carbohydrate ingestion (*p* < 0.05). PI3K and p70S6K activation were both elevated after exercise in both conditions (*p* < 0.05). However, AMPK activity did not change from baseline in both conditions (*p* > 0.05). We conclude that FAK does not induce mTOR activation through PI3K crosstalk in response to exercise alone. In addition, FAK may not be regulated by AMPK catalytic activity, but this needs further research. Interestingly, carbohydrate-induced insulin signaling appears to activate FAK at the level of IRS-1 but did not enhance mTOR activity 1 h post-exercise greater than the placebo condition. Future research should investigate these interactions under different conditions and within different time frames to clearly understand the interactions between these signaling molecules.

## 1. Introduction

There is an important link between carbohydrate supplementation and performance which has led to a large degree of research being conducted in attempts to maximize exercise performance, enhance skeletal muscle anabolic signaling, and accelerate recovery [1]. Intramuscular glycogen and glucose serve as major glycolytic fuel sources during resistance exercise and if their availability is limited then performance can be hindered [2,3]. In addition to serving as an exercise energy substrate, intramuscular glycogen and glucose act as metabolic moderators of anabolic signaling in the post-exercise state [4,5,6,7,8]. More specifically, high levels of intramuscular glycogen and glycolytic flux enhance mammalian target of rapamycin complex one (mTOR) anabolic signaling, while lower concentrations stimulate muscle autophagy in cells, indicating carbohydrate availability influences protein turnover [4,5,6,7,8]. Large decreases in intramuscular glucose and glycogen content post-exercise, that are not replenished through diet, could blunt anabolic signaling via glycolytic nutrient sensitive regulation of mTOR [5,6,7,8,9]. In addition to serving as a metabolic regulator of anabolism, carbohydrate ingestion stimulates insulin release, which subsequently leads to the activation of mTOR through the insulin receptor substrate-1 (IRS-1), phosphatidylinositol 3-kinase (PI3K), and protein kinase B (AKT) signaling pathway [10]. The heterotrimer, 5′ AMP-activated protein kinase (AMPK), inhibits mTOR activity during the heightened metabolic stress of exercise by increasing tuberous sclerosis protein 1 (TSC1) and TSC2 GTPase-activating protein activity [11]. Insulin signaling stimulates glucose transporter protein type-4 (GLUT-4) translocation, leading to increased intramuscular glycogen concentrations post-exercise, augmenting mTOR signaling by increasing glycogen binding to AMPK’s β regulatory and inhibiting its kinase activity [12,13,14]. Taken together, carbohydrate supplementation can facilitate anabolic activation of mTOR through multiple means in the post- exercise state.

In conjunction with the metabolic stress, tension is frequently placed upon skeletal muscle during exercise leading to integrin and subsequent mTOR activation [15]. Integrins are heterodimeric transmembrane proteins containing intracellular tails, an extracellular stalk attached to a ligand binding head that has been implicated in sensing mechanical tension [15,16,17,18]. When integrins bind to extracellular matrix proteins, they become fully activated, stimulating integrin clustering and the formation of focal adhesion complexes around the intracellular tails [16,17,18,19,20]. Tails of activated integrins do not possess enzymatic activity and require proteins within the focal adhesion complex to carry out the initial steps of mechanotransducive signaling for cellular adaptation. One of the main signaling proteins implicated in the response to mechanical strain is focal adhesion kinase (FAK) [21]. FAK is a cytoplasmic tyrosine receptor kinase that is subdivided into the N-terminal 4.1, ezrin, radixin, moesin homology (FERM) domain, C-terminal focal adhesion targeting domain, and kinase domain [22]. Within the linking connection between the FERM and kinase domain, the Tyr^397^ residue has shown to become auto-phosphorylated upon integrin activation from mechanical strain and enables Tyr^576^ and Tyr^577^ to become phosphorylated [23,24]. The phosphorylation of Tyr^397^ has been proposed to be the first step that allows FAK’s kinase domain to become catalytic activity through a series of Tyr phosphorylations [25,26]. FAK’s kinase domain may directly inhibit tuberous sclerosis complex 2 (TSC2), thereby increasing mTOR activation in response to FAK activation [27]. However, there is a second proposed route of FAK signaling that has been less researched but has been speculated to influence mTOR activity. Phosphorylation of FAK Tyr^397^ creates a binding site for SH2 domain containing proteins such as proto-oncogene tyrosine-protein kinase Src (Src) and PI3K [24,28,29]. In cell culture and animal models, PI3K has been shown to bind FAK Tyr^397^ leading to increased phosphatidylinositol (3,4,5)-trisphosphate (PIP3) signaling, while FAK inhibition has been shown to impair AKT and mTOR activity, which suggests an alternate link to mTOR signaling [28,29,30,31,32]. These studies may lead one to speculate that exercise-mediated FAK activation can lead to PI3K binding and activation of mTOR signaling. However, this alternate route of mTOR activation through FAK and PI3K to AKT is understudied in skeletal muscle and needs further elucidation.

In addition to serving as a biochemical transducer for tension, FAK has been implicated as a key metabolic signaling molecule. It appears that FAK is a required substrate in the insulin signaling cascade and may influence insulin-mediated mTOR activation [33,34,35,36]. Interestingly, AMPK activation was previously shown to be related with decreased FAK Tyr^397^ phosphorylation in isolated skeletal muscle samples potentially indicating FAK may be responsive to metabolic states in a similar manner as mTOR [14,37,38]. Clearly, there is a large degree of crosstalk between FAK, insulin, and AMPK signaling in the regulation of mTOR activity. Taken together, these nutrient, metabolic, and mechanical load-related signaling proteins appear to interact with each other to regulate the overall signaling cascade that is occurring within skeletal muscle cells in response to metabolic stress, diet, and exercise mechanical loading. Therefore, the purpose of this study was to investigate the activation of FAK, IRS-1, PI3K, AMPK, and p70S6K before and 1 h following a single bout of resistance exercise with pre-exercise carbohydrate consumption compared to a placebo condition. Specifically, we aimed to investigate if mTOR activity, measured as p70S6k Thr^389^ phosphorylation, was greater with both exercise and carbohydrate consumption due to elevations of both FAK and insulin mediated activation of PI3K activity compared to a resistance exercise alone. We hypothesize that FAK will increase PI3K catalytic activity in response to exercise and the combination of carbohydrate supplementation and exercise would lead to additional signaling through the PI3K pathway leading to increase p70S6K phosphorylation. We also aimed to investigate whether AMPK catalytic activity negatively influences FAK activity during acute resistance exercise with or without carbohydrate consumption.

## 2. Materials and Methods

Ten healthy, recreationally resistance-trained men (regular (i.e., thrice weekly) for at least 1 year), who had a mean ± standard deviation (SD) body mass of 90 ± 18.2 kg, height 179.2 ± 5.7 cm, age 21.6 ± 2.27 years, 5.98 ± 1.55 leg press strength-to-body weight ratio, 525.9 ± 5.8 kg leg press 1-repitition maximum (1-RM), and body fat 21.3 ± 8.1%, participated in this study. Participants that were surveyed reported an average training age of 5.8 ± 3.7 years, a weekly lifting frequency of 4.5 days/week, and average training time of 7.9 ± 2.9 h/week. When asked the goals of their training many reported that their training goal included both strength and hypertrophy outcomes. This study was approved by the Institutional Review Board for Human Subjects at Baylor University (project identification number 1335087-4). Additionally, all experimental procedures involved in the study conformed to the ethical consideration of the Declaration of Helsinki. A detailed description of the resistance exercise protocol, dietary controls, and supplementation protocol can be seen in our previously published manuscript [39].

Briefly, during the first visit, participants were familiarized to the study protocol via a verbal and written explanation outlining the study design and then read and signed a university-approved informed consent document. In addition, each participant was instructed to refrain from exercise for 48 h before each testing session, arrive 3 h fasted prior to reporting for each testing session, and record their dietary intake for two days (including the light meal the morning of testing) prior to each of the three testing sessions. Diets were not standardized but participants were asked not to change their dietary habits. The MyFitnessPal mobile application (Under Armor Inc., Baltimore, MD, USA) was used to determine average daily macronutrient consumption of fat, carbohydrate, and protein. Participants completed a medical history and exercise questionnaire and underwent a general physical examination to determine whether they further met eligibility criteria.

During session 2, participants performed angled leg press one-repetition maximum (1-RM) tests with the National Strength and Conditioning Association (NSCA) recommendations [40]. Foot placement was recorded and held constant over all testing conditions. A goniometer was used to establish 90° of knee flexion while positioned on the leg press machine and safety catches were adjusted just below this point for all tests to standardize the range of motion to 90°. The participants were instructed to lower the weight just above the catches before pressing the weight upward. Participants warmed up with 10 repetitions at approximately 50% estimated 1-RM, rested 1 min, and completed five repetitions at approximately 70% estimated 1-RM followed by 2 min rest. The weight was then increased conservatively, and the participants attempted the first 1-RM. If the lift was successful, the participant rested for 2 min before attempting the next 1-RM. If the perceived exertion of a subsequent 1-RM attempt was below 6 on the Borg Scale, then the load was increased between 20 to 40 lbs. However, if the perceived exertion of a prior 1-RM was 6 or above then loads were increased between 5 to 15 lbs accordingly. The 1-RM of each participant was compared to male 90% rank normative values of age specific strength-to-body weight ratios (2.27) [41]. This substantiated that all participants were trained for at least a year as stated in the exercise questionnaire. Thirty minutes following the 1-RM test, participants completed four sets of maximal repetitions as outlined in the resistance exercise protocol below.

Each participant reported to sessions 3 and 4 at the same time one week apart to control for time of day variations within subject measurements. During testing visits 3 and 4, in a randomized, double-blind fashion, participants ingested either a placebo or carbohydrate supplement (2 g/kg) 30 min before completing four sets of repetitions to volitional fatigue with 70% of the 1-RM on the angled leg press. Percutaneous muscle biopsies (~30 mg each obtained from 2–3 needle passes) were completed at both testing visits using fine needle aspiration prior to exercise before supplementation and 1 h post-exercise. Biopsies were obtained from the middle portion of the vastus lateralis muscle of the dominant leg at the midpoint between the patella and the greater trochanter of the femur at a depth between 1 and 2 cm. After the initial biopsy, remaining biopsy attempts were made to extract tissue from approximately the same location as the initial biopsy using the pre-biopsy scar and depth markings on the needle, and a successive puncture was made approximately 0.5 cm from medial to lateral. Adipose tissue was trimmed, and muscle specimens were immediately stored at −80 °C for later analysis. 

Activated human PI3K catalytic subunit-α isoform (MyBioSource, San Diego, CA, USA), activated AMPK phosphorylated at α-subunit Thr^172^ (Thermofisher Scientific, San Francisco, CA, USA), activated FAK phosphorylated at Tyr^397^ (Thermofisher Scientific, San Francisco, CA, USA), activated p70S6K phosphorylated at Thr^389^ (Cell Signaling Technology, Danvers, MA, USA), and activated IRS-1 (^pan^Tyr; Cell Signaling Technology, Danvers, MA, USA) were assessed using commercially-available enzyme-linked immunosorbent assay kits. The absorbances of all variables were determined with a microplate reader (X-Mark, Bio-Rad, Hercules, CA, USA) and concentrations of PI3K, AMPK, and FAK were determined by linear regression against known standard curves using commercial software (Microplate Manager, Bio-Rad, Hercules, CA, USA) as per the manufacturer’s instructions. All absorbances and concentrations for each variable were made relative to the total protein content within the sample time point. The analysis was conducted on the pre-exercise and 1 h post-exercise time points based on previous literature indicating that insulin, FAK, mTOR, and AMPK signaling could be simultaneously occurring at the 1 h time point [39,42,43,44]. All samples were assayed in duplicate and the average ± SD intra-assay coefficients of variation for FAK, p70S6K, IRS-1, AMPK, and PI3K were 4.0 ± 3.5%, 4.35 ± 6.5%, 5.45 ± 4.9%, 5.9 ± 5.4%, and 5.5 ± 6.8%, respectively. The manufacturers describe the sensitivity of the FAK, p70S6K, IRS-1, AMPK, and PI3K ELISA kits to be <0.09 Units/mL of FAK phosphorylated at Tyr^397^, 0.031 mg/mL of p70S6K phosphorylated at Thr^389^, 0.018 mg/mL of activated IRS-1 (^pan^Tyr), <1 Unit/mL AMPKα-subunit phosphorylation at Thr^172^, and <0.094 ng/mL PI3K catalytic subunit-α isoform, respectively. 

All statistics were analysis were conducted using the commercially available software IBM SPSS Statistics for Windows, version 25 (IBM Corp., Armonk, NY, USA). To test the normality of the data we analyzed the studentized residuals using visual analysis of Q-Q plots and completed a Shapiro–Wilks tests for each independent variable condition. The studentized residuals displayed a normal distribution meeting the normality requirements. Subsequently, for each of the variables a 2 × 2 (Condition (CHO, Placebo) × Time (pre-exercise, 1 h post- exercise)) factorial analyses of variance (ANOVA) with repeated measures was conducted at a significance level of 0.05. If a significant main effect was found, then pairwise comparisons with a Bonferroni adjustment was completed to assess the difference. If a significant interaction was found, then simple effects analysis with a Bonferroni adjustment were completed to assess where the interaction occurred. In addition, dietary recalls were analyzed using a separate one-way repeated measures ANOVA for each macronutrient with a significance level of 0.05. Partial Eta squared (η^2^), was used to estimate the proportion of variance in the dependent variable explained by the independent variable for each conducted ANOVA. Partial Eta squared effect sizes determined to be: weak = 0.17, medium = 0.24, strong = 0.51, or very strong = 0.70.

## 3. Results

### 3.1. Macronutrient Intake

Upon analysis of the 48-h dietary recall for each testing visit, there was no statistical difference between fat (F = 0.087, *p* = 0.917, η^2^ = 0.01), carbohydrate (F = 1.051, *p* = 0.370, η^2^ = 0.105), or protein (F = 0.576, *p* = 0.572, η^2^ = 0.06) intake. Table 1 below shows the means and standard deviations of each macronutrient that was consumed before each visit.

### 3.2. FAK Tyr^397^ Phosphorylation

**Figure 1:**
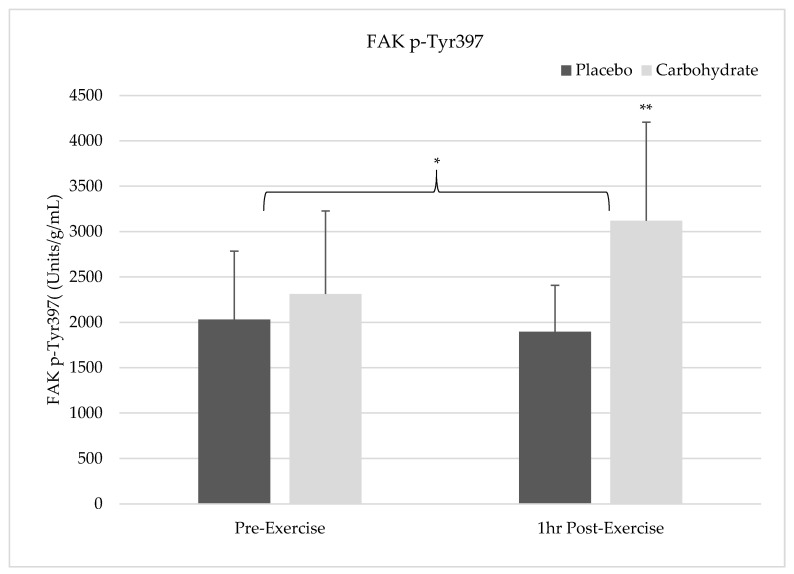
Changes in focal adhesion kinase (FAK) phosphorylation at Tyr^397^ for each time point and condition relative to total protein content are illustrated. There was a significant interaction showing a greater increase in FAK Tyr^397^phosphorylation 1 h post-exercise (**). In addition, there was a significant condition effect showing carbohydrate supplementation increased FAK Tyr^397^ phosphorylation greater than the placebo condition (*).

There was a time by condition interaction showing FAK activation was significantly higher 1 h post-exercise in the carbohydrate group (3118 Units/g/mL ± 1086) compared to the placebo condition (1897 Units/g/mL ± 510; F = 5.844, *p* = 0.042, η^2^ = 0.422). There was also a main effect for condition showing CHO (2714 Units/g/mL ± 1001) had significantly higher FAK activation across both time points compared to placebo (1964 Units/g/mL ± 631; F = 6.026, *p* = 0.04, η^2^ = 0.43). However, there was no main effect for time on FAK activation between pre-exercise (2171 Units/g/mL ± 0.835) and 1 h post-exercise (2507 Units/g/mL ± 288; F = 0.634, *p* = 0.452, η^2^ = 0.072). The changes in FAK can be seen in Figure 1 below.

### 3.3. p70S6K Thr^389^ Phosphorylation

**Figure 2:**
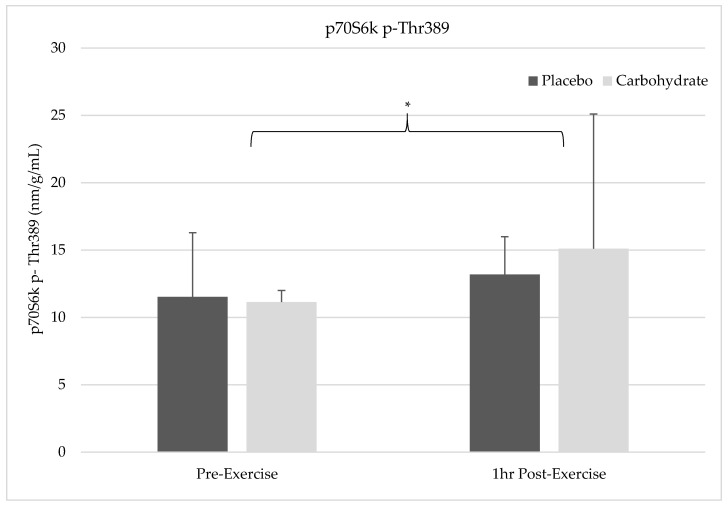
Changes in total amount of ribosomal protein S6 kinase beta-1 (p70S6K) phosphorylated at Thr^389^ for each timepoint and condition relative to total protein content are illustrated. There was a significant time effect showing increases in p70S6K Thr^389^ phosphorylation from pre-exercise to 1 h post-exercise (*).

There was not a time by condition interaction for p70S6K activation (F = 0.850, *p* = 0.384, η^2^ = 0.096). There was also no main effect for supplement condition on p70S6K activation between placebo (12.4 nm/g/mL ± 3.78) and CHO (13.1 nm/g/mL ± 1.71) conditions (F = 0.953, *p* = 0.358, η^2^ = 0.106). However, there was a significant main effect for time showing increases in p70S6K activation in both placebo and CHO from pre-exercise (11.3 nm/g/mL ± 2.8) to 1 h post-exercise (14.1 nm/g/mL ± 2.8; F = 6.67, *p* = 0.032, η^2^ = 0.455). The changes in p70S6K can be seen in Figure 2 below. 

### 3.4. IRS-1 (^pan^Tyr) Phosphorylation

**Figure 3:**
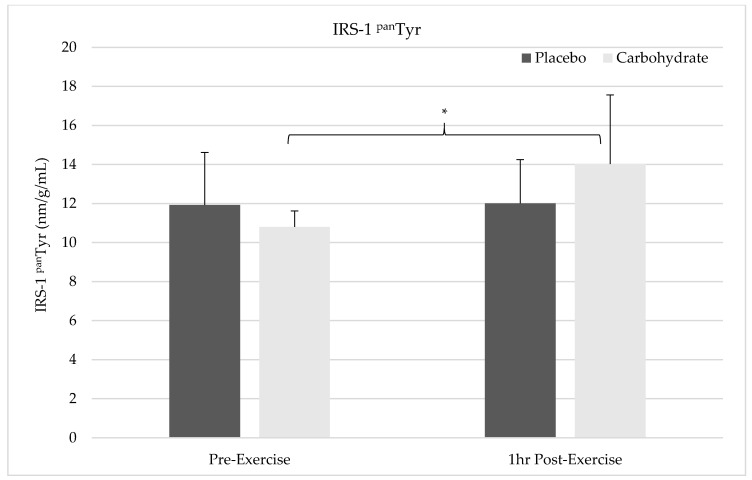
Changes in total amount of activated insulin receptor substrate-1 (IRS-1) ^pan^Tyr for each time point and condition relative to total protein content are illustrated. There was an interaction effect showing a significant increase in IRS-1 phosphorylation in the carbohydrate condition at 1 h post-exercise compared to the placebo (*). No change in IRS-1 (^pan^Tyr) was observed for the placebo condition.

There was a time by condition interaction effect showing a significant increase in IRS-1 activation in the carbohydrate condition at 1 h post-exercise (13.2 nm/g/mL ± 3.4) compared to the placebo (12.0 nm/g/mL ± 2.2; F = 5.323, *p* = 0.05, η^2^ = 0.40). Simple effects analysis indicated carbohydrate supplementation increased IRS-1 activation at the 1 h post-exercise time point and 40% of the variation in this measure was due to the supplementation protocol (*p* = 0.011). There was no main effect for time indicating average IRS-1 activation at pre-exercise (13 nm/g/mL ± 4.79) and 1 h post-exercise (12.6 nm/g/mL ± 2.8) were the same (F = 2.424, *p* = 0.158, η^2^= 0.233). There was no significant condition effect for IRS-1 activation between placebo (11.9 nm/g/mL ± 2.5) or carbohydrate (13.8 nm/g/mL ± 5.16) conditions (F = 0.068, *p* = 0.80, η^2^ = 0.001). Figure 3 shows the changes in IRS-1 (^pan^Tyr) phosphorylation below. 

### 3.5. AMPK α-Subunit Thr^172^ Phosphorylation

There was not a significant time by condition interaction between time and condition on AMPK activation (F = 0.031, *p* = 0.864, η^2^ = 0.004). There was not a significant main effect for time on AMPK activation between pre-exercise (476 Units/g/mL ± 311) or 1 h post-exercise conditions (703 Units/g/mL ± 348; F = 2.212, *p* = 0.175, η^2^ = 0.217). There was not a significant a significant difference in AMPK activation between placebo (577 Units/g/mL ± 286) or carbohydrate conditions (602 Units/g/mL ± 372; F = 0.056, *p* = 0.819, η^2^ = 0.007). 

### 3.6. PI3K Catalytic Subunit-α Isoform

**Figure 4:**
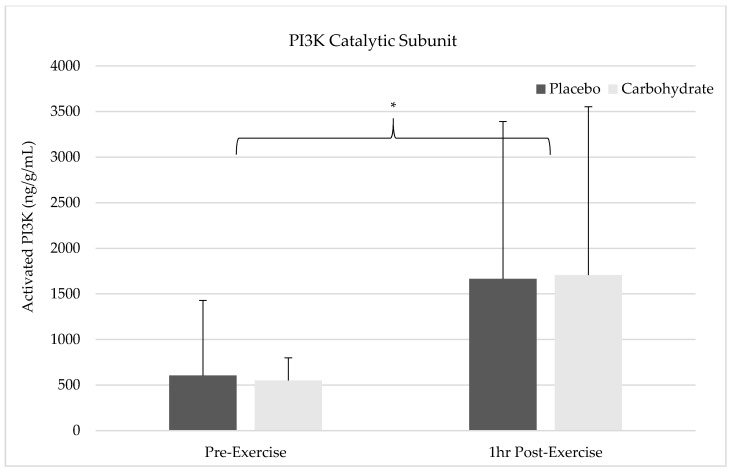
Changes in total amount of activated phosphatidylinositol 3-kinase (PI3K) p110 subunit in for pre-exercise and 1 h post-exercise with or without carbohydrate supplementation relative to total protein content are illustrated. There was a significant increase in PI3K activation across time for both conditions (*).

There was not a significant time by condition interaction on PI3K activation (F = 0.014, *p* = 0.909, η^2^ = 0.002). There was not a significant main effect for condition between placebo (1135 ng/g/mL ± 1273) or carbohydrate (1128 ng/g/mL ± 1048) on activated PI3K (F = 0.008, *p* = 0.932, η^2^ = 0.001). There was a significant main effect for time showing and increase in PI3K activation from pre-exercise (577 ng/g/mL ± 536) to 1 h post-exercise (1686 ng/g/mL ± 1785; F = 9.672, *p* = 0.014, η^2^ = 0.547). The alterations in PI3K activation can be seen in Figure 4 below. 

## 4. Discussion

The results of this study show pre-exercise carbohydrate supplementation can induce the activation of IRS-1 and PI3K but will not synergistically elevate p70S6K Thr^387^ phosphorylation compared to the resistance exercise alone. Despite FAK’s capacity to form an SH2 binding domain by Tyr^397^ phosphorylation, it appears that FAK and PI3K do not crosstalk in response to exercise since PI3K activity was elevated without simultaneous FAK activation. FAK was not shown to be activated 1 h post-exercise in the placebo condition and future researcher should carefully consider the sampling time frames when attempting to measure activity of this non-receptor tyrosine kinase. Carbohydrate-induced insulin signaling appears to activate FAK through crosstalk at the level of IRS-1, indicating integrative role of nutrition and exercise between these molecules. Interestingly, AMPK catalytic activity was not elevated in either the placebo or carbohydrate conditions indicating it was not responsible for FAK inhibition. These findings suggest that carbohydrate is not the main macronutrient regulator of mTOR activity but may have a role in regulating or integrating the mechanotransducive response from resistance exercise.

Carbohydrates are understood to be a critical source of fuel for exercise performance and recovery [1]. In addition, intramuscular carbohydrate content has been shown to serve as a metabolic regulator of mTOR within skeletal muscle [4,5,6,7,8]. Cell culture studies that have indicated a direct regulation of mTOR activity by glycolytic enzymes implemented a glucose deprivation method that starved the cell of carbohydrates [6,7,8]. This may be a state that is unobtainable to be implemented for observation in human studies unless an individual’s wellbeing is severely compromised. Nevertheless, we observed no difference in p70S6K Thr^389^ phosphorylation between carbohydrate and placebo conditions. Carbohydrate can induce anabolic signaling through the insulin signaling pathway to mTOR, subsequently increasing p70S6K activation in response to increased nutrient availability [10]. Resistance exercise in the fed state after a mixed meal has shown positive synergistic effects on p70S6K activation [45]. Conversely, previous research indicated that a drink consisting of both protein and carbohydrate did not stimulate synergistic increases in muscle protein synthesis compared to a protein only drink despite seeing greater elevations of plasma insulin and intramuscular AKT phosphorylation with carbohydrate [46]. These findings [46] indicate that protein serves as the main nutrient regulator of mTOR while carbohydrate serves a secondary role in potentiating anabolism. Our results support these findings [46] since no difference in p70S6K Thr^389^ phosphorylation between carbohydrate and placebo conditions was observed, despite seeing increased intramuscular insulin signaling. This indicates carbohydrate mediated insulin signaling can further facilitate the switch from catabolic to anabolic states but serves as a secondary contributor of mTOR signaling compared to protein [4,6,7,8,45,46]. The regulation of mTOR has been shown to be dependent on nutrient availability, mechanical loading, cellular metabolic state, and endocrinological stimulation [4,5,8,10,35,47]. During the recovery period post-exercise, these variables coordinate the signaling cascade that is mounted within the cell to produce the needed proteins for adaptation. These biomolecular pathways have been shown to interact and regulate each other in a complex manner that is still being studied. To understand if mechanical load-induced FAK activation alters mTOR activity through crosstalk with PI3K, we measured FAK Tyr^397^ phosphorylation before and 1 h post-exercise with or without carbohydrate supplementation. If elevations in Tyr^397^ were seen it would increase the likelihood for PI3K to bind and become active, thereby inducing PI3K, AKT, and mTOR activity. Other studies have shown potential for FAK to bind PI3K’s p85 regulatory subunit and activate it as a potential crosstalk mechanism that could influence mTOR [29]. However, we did not observe elevations in FAK Tyr^397^ phosphorylation in the placebo condition indicating that this binding site was not available 1 h post-exercise for FAK-PI3K crosstalk. Interestingly, our results show increases in PI3K catalytic activity and p70S6K Thr^389^ phosphorylation in the placebo condition despite not observing FAK activity. Since FAK Tyr^397^ phosphorylation was unchanged in the placebo condition, the alternative FAK Tyr^576/577^ activating mTOR pathway through TSC2 was not active since Tyr^397^ phosphorylation is a pre-requisite for initiation of that signaling cascade [27]. It is conceivable that the increased p70S6K Thr^389^ phosphorylation likely occurred through the PI3K-AKT signaling pathway, without solely being contingent on increases in FAK activity. It may be possible that other mechanotransductive elements independent from FAK, perhaps calcium signaling, increased PI3K catalytic activity thus increasing p70S6K Thr^387^ phosphorylation in the placebo condition [48,49]. Overall, our results indicate that FAK does not crosstalk directly with PI3K, and mTOR activation can be induced by other mechanisms independent of FAK.

In addition to FAK-mediated mTOR signaling in our placebo condition, we sought to determine if there was an enhancement of mTOR activity with insulin signaling that was stimulated by carbohydrate ingestion. Since FAK was not activated in the placebo condition 1 h post-exercise, it is difficult to determine if exercise-induced FAK activation occurred and if there were enhanced insulin signaling effects on mTOR. Positive synergistic effects on mTOR activity have previously been found in other studies utilizing both feeding and exercise; however, we did not see additive effects on p70S6K Thr^389^ phosphorylation despite carbohydrate ingestion pre-exercise [45]. Considering this, a compounding effect on p70S6k activation from mechanical load and carbohydrate-induced insulin signaling could not be determined from the present study. This lack of an effect may have been due to the sampling time frame that was used. FAK activation has been shown to be significantly elevated immediately post-exercise and after muscle massage and returns to baseline values within 3–4 h [44,50]. In our placebo condition, FAK activation was not observed, indicating that this window of activation is shorter than 1 h post-exercise. Another study with a similar protocol indicated that four sets of 10 repetitions on leg press and leg extensions did not result in FAK phosphorylation [45]. However, the biopsy sampling time frame for the study was pre-exercise and 6 h post-exercise, well after the suspected 3–4 h window when FAK activation has been reported by other studies [44,45,50]. Future research would likely benefit viewing these signaling molecules immediately post-exercise or within 30 min of the exercise bout to ascertain a viable sampling time frame.

Despite the lack of FAK activation in the placebo condition, FAK Tyr^397^ phosphorylation in the carbohydrate condition was elevated suggesting carbohydrate-mediated insulin signaling stimulates the activation of FAK. Our results align with previous findings suggesting there is an occurrence of crosstalk between FAK and the insulin signaling pathway [33,35,51,52]; however, this mechanism in skeletal muscle remains unclear. In cell culture, IRS-1 was previously co-immunoprecipitated with FAK without requiring FAK catalytic activation and was suggested to use IRS-1 as a docking protein allowing crosstalk between integrin and insulin signaling pathways [52]. IRS-1 phosphorylation was stimulated by FAK but only when an active Src was bound to the Tyr^397^ residue, indicating mechanical loading or cellular attachment facilitates this interaction [52]. Another cell culture study used small interfering RNA to block FAK activation and found IRS-1 phosphorylation was inhibited even with insulin administration supporting the idea of IRS-1-FAK crosstalk and a required roll of FAK in insulin signaling [33]. Our results also suggest that FAK and IRS-1 are the proteins of crosstalk that regulate each signaling pathway however, our results indicate that insulin activation of IRS-1 can mediate FAK Tyr^397^ phosphorylation. It is plausible that load-induced FAK signaling and carbohydrate-induced insulin signaling pathways co-regulate each other in various situations within skeletal muscle to mediated mTOR activity in response to exercise and nutrient availability. Our results directly show pre-exercise ingestion of carbohydrate augments the mechanotransductive responsiveness of skeletal muscle through interactions with FAK. We are limited to interpreting these findings within the time points that we sampled but carbohydrate ingestion may extend the duration of mTOR signaling by activation of multiple agonistic pathways. Longer time frames in future research are needed to investigate these responses to determine if carbohydrate can mediate a greater area under the curve measure for p70S6K activity compared to resistance exercise alone.

Our results also indicate there was no change in AMPK catalytic activation 1 h post-exercise for either supplement condition. Previous research has found elevations in AMPK catalytic activity 1 h post-exercise after participants completed 10 sets of 10 repetitions of leg extensions at 70% of their 1-RM with 3 min inter-set rest periods [43]. Although this study used the same 1-RM percentage, there is a distinct difference in the completed number of reps between their study (100 repetitions) and our own (52.8 ± 7.3 repetitions) [43]. Additionally, participants underwent a standardized overnight fast lasting 10 h before completing the exercise and recovery protocol which lasted an additional 6 h after waking [43]. Participants had not eaten for 15 h and 16 h, at the 1 h and 2 h post-exercise sampling time points, while our fasting period was only limited to 3 h before the testing session [43]. The combination of a longer fasting period and greater exercise volume likely led to difference in AMPK activity between that study and our own [43]. However, AMPKα Thr^172^ phosphorylation was previously shown to not be significantly elevated 1 h post-exercise in young individuals compared to older individuals after completing eight sets of 10 repetitions (80 repetitions) of leg extensions at 70% 1-RM with 3 min inter-set rest periods after an overnight fast [53]. This study utilized a similar methodology and fasting protocol as the methods described by [43] but saw no elevations in AMPK activity at similar time points post-exercise further indicating volume of the exercise bout can directly influence AMPK activity [53]. In our study, our exercise protocol did not elicit a robust AMPK response due to the lower exercise volume that was completed. In a more comparable study, AMPK catalytic activity was shown to be elevated immediately post-exercise, when participants completed five sets of 8–10 repetitions at 80% of 1-RM, which returned to basal values 4 h after resistance exercise with feeding [44]. This difference in 1-RM percentage may also have led to the varied results found between studies however, we cannot rule out the possibility that AMPK activity was elevated immediately post-exercise declined to basal values at 1 h post-exercise in our study. In addition, this study found that FAK Tyr^576/577^ phosphorylation was elevated immediately post-exercise and did not appear to be influenced by AMPK catalytic activity [44]. Collectively, our results and those of the aforementioned studies, indicate controversial findings of AMPK activity post-exercise that seem to be mediated by volume, load, fasting, and age [43,44,53]. In addition, FAK activity may not be directly mediated by AMPK since its inhibition was not correspondingly related to AMPK activation. Our results and other in vivo studies question the regulatory role that AMPK may have on FAK activation that was established in cell culture and requires further investigation [37,44]. Additionally, these inconsistent findings of AMPK activity in response to exercise warrant further investigation with distinct controls for the indicated variables outlined above.

One of the main limitations of our study was the time sampling utilized in the analysis. The 1 h post-exercise time point may have been too late to see AMPK or FAK activation post-exercise in the placebo condition. If there is AMPK or FAK activation immediately post-exercise, this could influence the acute responses of the signaling proteins measured in this study. However, from this limitation our results give indication of a potential brief window for observing the activation of these signaling molecules in response to mechanical loading. In addition, this limitation also indicates potential intracellular signaling redundancies that leads to increases in mTOR activation without FAK activation post-exercise. In addition, our sample was specifically comprised of a smaller group of healthy trained male participants which may limit the translatability of these findings into other populations. The results of this study may not reflect the exact signaling cascade that could occur in untrained, female, or clinical populations. Translation of the findings into another populations warrants caution. In addition, we did not completely standardize diet for each participant to eat prescribed meals and variations in self-reporting may slightly influence the results. Despite this limitation, no significant differences were found between the testing visits for any macronutrients indicating that diet was controlled for.

## 5. Conclusions

The initial aim of this study was to investigate the relationship between nutritional and mechanical responsive intracellular signaling molecules and how they can regulate mTOR activation. Apparently, exercise and pre-exercise carbohydrate supplementation does not exacerbate the anabolic mTOR signaling that is initiated after resistance exercise. These results indicate that recreationally trained individuals, eating a normal diet, do not need to supplement with excess carbohydrates after resistance exercise since it will not dramatically increase mTOR activity despite increased insulin signaling. Lack of FAK activity in response to exercise alone was not attributed to AMPK activation and needs further investigation under different exercise volumes, loads, and durations. Additionally, we show brief resistance exercise to fatigue coupled with carbohydrate consumption initiates crosstalk between IRS-1 and FAK. This crosstalk demonstrates skeletal muscles ability to incorporate both mechanical load and nutritional signals into similar signaling cascades that potentially mediate mTOR activity. Further research is needed to investigate the mechanisms by which these signaling molecules function and possibly cooperatively regulate each other in response various exercise scenarios. Based on the results herein, we conclude that pre-exercise carbohydrate ingestion will subsequently lead to increased insulin signaling and nutrient-mediated FAK activation but not larger increases in mTOR stimulated p70S6K phosphorylation compared to a placebo.

## Figures and Tables

**Table 1:** Mean (± SD) macronutrient intake for all participants between the testing conditions.

Macronutrients	Preliminary Visit	Placebo	Carbohydrate
Carbohydrate (g/kg)	3.3 (±0.98)	2.8 (±1.18)	2.7 (±1.11)
Fat (g/kg)	1.3 (±0.50)	1.1 (±0.44)	1.08 (±0.48)
Protein (g/kg)	2.1 (±0.72)	1.5 (±0.38)	1.3 (±0.59)
